# Differentiation of haploid and diploid fertilities in *Gracilaria chilensis* affect ploidy ratio

**DOI:** 10.1186/s12862-018-1287-x

**Published:** 2018-12-05

**Authors:** Vasco M. N. C. S. Vieira, Aschwin H. Engelen, Oscar R. Huanel, Marie-Laure Guillemin

**Affiliations:** 1 MARETEC, Instituto Superior Técnico, Universidade Técnica de Lisboa, Av. Rovisco Pais, 1049-001 Lisbon, Portugal; 2 CCMAR, Center of Marine Science, University of Algarve, Campus Gambelas, 8005-139 Faro, Portugal; 3 Instituto de Ciencias Ambientales y Evolutivas, Facultad de Ciencias, Universidad Austral de Chile, Casilla 567, Valdivia, Chile; 4 Departamento de Ecología, Facultad de Ciencias Biológicas, Pontificia Universidad Católica de Chile, Casilla 114, -D Santiago, Chile; 5 CNRS, Sorbonne Universités, UPMC University Paris VI, UMI 3614, Evolutionary Biology and Ecology of Algae, Station Biologique de Roscoff, CS 90074, Place G. Tessier, 296888, Roscoff, France

**Keywords:** Gametophyte, Tetrasporophyte, Isomorphic biphasic life cycle, Phase ratio, Ploidy ratio, Recruitment

## Abstract

**Background:**

Algal isomorphic biphasic life cycles alternate between free-living diploid (tetrasporophytes) and haploid (dioicious gametophytes) phases and the hypotheses explaining their maintenance are still debated. Classic models state that conditional differentiation between phases is required for the evolutionary stability of biphasic life cycles while other authors proposed that the uneven ploidy abundances observed in the field are explained by their cytological differences in spore production.

**Results:**

We monitored the state and fate of individuals of the red seaweed *Gracilaria chilensis* periodically for 3 years in five intertidal pools from two sites with distinct conditions. We tested for differentiation in fecundity and spore survival among the gametophyte males and females (haploids) and the tetrasporophytes (diploids). We tested for the influence of fecundity and spore survival on the observed uneven ploidy abundances in recruits. The probability of a frond becoming fecund was size-dependent, highest for the haploid males and lowest for the haploid females, with the diploids displaying intermediate probabilities. Fecund diploids released more tetraspores than carpospores released by the haploid females. Spore survival depended on ploidy and on the local density of co-habiting adult fronds. An advantage of diploid over haploid germlings was observed at very low and very high adult fronds densities.

**Conclusions:**

Neither spore production nor spore survival determined the highly variable ploidy ratio within *G. chilensis* recruits. This result invalidates the hypothesis of natural cytological differences in spore production as the only driver of uneven field ploidy abundances in this species. Diploid spores (carpospores) survived better than haploid spores (tetraspores), especially in locations and time periods that were associated with the occurrence of strong biotic and abiotic stressors. We hypothesise that carpospore survival is higher due to support by their haploid female progenitors passing-on nutrients and chemical compounds improving survival under stressful conditions.

**Electronic supplementary material:**

The online version of this article (10.1186/s12862-018-1287-x) contains supplementary material, which is available to authorized users.

## Introduction

An alternation between haploid and diploid nuclear phases is a necessary consequence of eukaryotic sexuality. However, due to variation in the relative timing of meiosis and syngamy in life cycles, the duration of these two phases vary widely among organism [1]. Although the multicellular haploid generation went extinct in the life cycle of vascular plants roughly 400 million years ago [2, 3], it persists in the haploid-diploid life cycles (also known as biphasic life cycles) commonly found, for example, in many green, red and brown seaweeds [1]. In these life cycles, free-living haploid gametophyte and diploid sporophyte phases are morphologically distinct from one another (heteromorphic) to seemingly identical to each other (isomorphic). In the heteromorphic case, gametophytes and sporophytes generally display marked differences in size, morphology and physiology, and have been observed to occupy different spatio-temporal niches (see for review Thornber [4]). Thus, it is not surprising that most developed hypotheses and models explaining the maintenance of haploid-diploid life cycles rely on the existence of some degree of niche differentiation between the two phases [5–7]. Mirroring the classical Lotka–Volterra model of interspecific competition [8, 9], the coexistence of haploid and diploid individuals in a population requires only slight differentiation in adaptation to different environments (i.e., differences in temperature, light levels or grazing pressure [7]).

In this context, the evolutionary stability of the isomorphic biphasic life cycle is complex to explain. Theoretically, two similar entities completely overlapping their niche cannot sustainably co-exist as one inevitably eliminates the other. However, the fact that phases in isomorphic biphasic life cycles have similar gross morphology does not necessarily mean that they are ecologically similar. Such ecological differentiation has been revealed in numerous cases during the last decades (e.g. [10–15]). Differences in chemical compounds have been shown to influence the fate of endophytic infection [16], palatability [17–20], tolerance to wave exposure [21] and desiccation [22]. So, these differences can cause profound differentiation in mortality and fertility rates between isomorphic phases. Even for isomorphic species for which no conspicuous biochemical differences have been revealed, ecological phase differences have been observed at both microscopic and macroscopic stages [13, 15, 23–25]. Therefore, as for heteromorphic life cycles, it has been argued that the evolutionary stability of isomorphic biphasic life cycles is sustained by the niche partitioning among ploidies, driven by the conditional differentiation of their individuals [7]. Many studies have reported uneven field abundances of haploids and diploids (i.e., commonly reported as the ratios of H:D or G:T, where H and G stands for the haploid gametophytes and D and T for the diploid tetrasporophytes). Furthermore, these uneven abundances often vary between species or even between populations of the same species [11–13, 15, 26–30]. Such differences in the haploid-diploid ratio have been associated to ecological dissimilarities between phases in fecundity, recruitment, growth or mortality [11, 12, 15]).

**Fig. 1:**
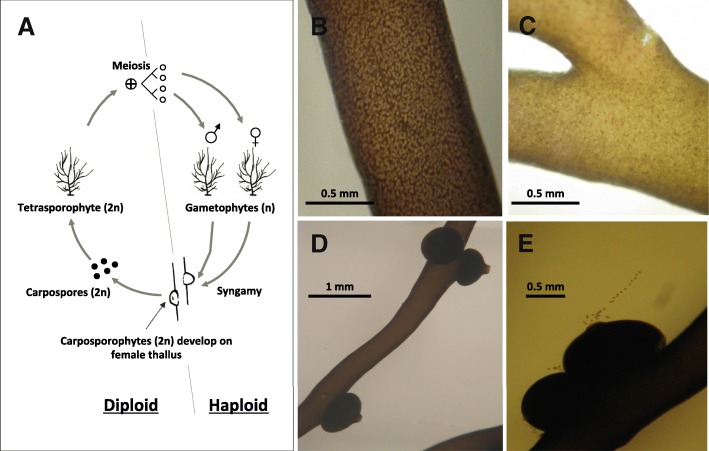
Isomorphic biphasic (haploid-diploid) life-cycle of *Gracilaria chilensis*. **a** Life cycle showing the free-living tetrasporophytes (diploids) and dioicious gametophytes (haploids, male and female) stages. Tetrasporophyte, when mature produce haploid tetraspores after meiosis and release them to the environment. Tetraspores settle and grow into adult male or female gametophytes. The gametophyte males release gametes that fertilize female gametophytes. From the fertilized oogonia, a short-lived diploid epiphytic stage (the carposporophyte) develops on the female thallus. Within each carposporophyte, the zygote undergoes successive mitoses allowing to produce many thousand of carpospores that are then released into the environment, where they settle and grow into diploid tetrasporopytes. **b** Mature male bearing gametocysts (i.e., ovate white spots). Male gametes are released in the water column. **c** Mature tetrasporophyte thallus with tetrasporocysts (i.e., structures where tetraspores are produced) visible. Deep red pigmented tetrasporocysts still contain tetraspores while round white spots correspond to empty tetrasporocysts. **d** Female thallus after fertilization bearing carposporophytes. **e** Detail of a carposporophyte liberating carpospores. All photographs by Paula Valenzuela

In red algae, the complex haploid-diploid life cycle is modified and includes a third phase: the carposporophyte (Fig. 1). In these organisms, the female gamete is retained on the thallus where fertilization occurs. The zygote undergoes successive mitotic divisions and develops into a multicellular carposporophyte, while remaining connected to the female thallus. Each carposporophyte can form thousands of carpospores that are, ultimately, released into the water. It has been postulated that this mitotic amplification of the zygote may have evolved to compensate for low natural fertilization success due to the lack of motile male gametes in this group [31]. However, Engel et al. [32] demonstrated that fertilization success in *Gracilaria gracilis* is not limited by the availability of male gametes or their lack of mobility. The production of tetraspores is considered to require only the growth of diploid tetrasporophytes, tetrasporangia development and tetraspore production by meiosis [33]. In contrast, carpospore production has to be preceded by the growth of both male and female haploid gametophytes, gametes production, fertilization, carposporophyte development, as well as carpospore production. Moreover, even though it has been hypothesized that algae invest few resources toward reproduction (i.e., no ancillary reproductive structures and all reproductive structures are photosynthetic [34]), the possibility of trade-offs in resources among survival, growth and reproduction have been postulated [15]. This is especially so for female gametophytes supplying nutrients for the development of cystocarps [35]. Because of these particularities, different fecundity rates may reasonably be expected for haploid gametophytes and diploid sporophytes in red algae, and have indeed been reported in a large number of species (e.g., [10, 12–14, 17, 19, 20, 24, 36, 37]). These empirical results have been proposed to support the models where the observed uneven field abundances of haploids and diploids emerge as a natural consequence of the differences in phase fertility without the necessity of any niche partitioning [12, 38, 39]. However, as is common in red algae, *Gracilaria gracilis* sheds both tetraspores and carpospores in abundance in nature [11]. Moreover, population persistence and growth were mostly driven by survival of the gametophyte and tetrasporophyte stages and it was postulated that differences in fertility could be ecologically irrelevant [11]. Indeed, theoretical works support that conditional differentiation of the traits related to survival rates, and not fertility, was most efficient to partition niches between phases in haploid-diploid organisms [40–43]. In any case, these hypotheses are not mutually exclusive and more complex models are emerging where the differences in fertility could explain the general trend in haploid-diploid ratio characterizing each species, while site specificity and spatial and seasonal variability can only result from conditional differentiation of the traits related to survival [12, 41].

If the survival and fertility of both phases is equivalent, demographic models predict that the proportion of diploids present in a haploid-diploid population at the equilibrium should be 0.41 [12, 44]. Though most *G. gracilis* populations studied showed diploid proportions close to 0.41 [11], this was not the case for the closely related species *G. chilensis* [45]. Indeed, in *G. chilensis* the proportion of diploids varies widely among populations – from clear excess of haploids to clear excess of diploids - with a tendency to show more populations with excess of diploids (7 of the 11 natural populations studied show a proportion of diploids > 0.41, [45]). Interestingly, in *G. chilensis*, the existence of a clear conditional differentiation of survival between phases was demonstrated in the field [15]. Haploid females were shown to survive more in stressful conditions (namely under very low or very high densities) than the haploid males and diploids and, when fertile, the survival of haploid females also surpassed the survival of the diploid tetrasporophytes [15]. In this case, differences in survival between phases did not seem to explain the generally high frequency of diploids observed in previous studies [45]. Differences in fertility or recruitment have not been thoughtfully tested yet in the field for this species, but previous experiments in controlled laboratory conditions have, though, given some insight in the existence of possible phase differences for spore attachment and survival and growth of germlings [25]. Here, using natural populations of *G. chilensis* monitored for 28 months, we test for field evidences of differences in fertility and recruitment among haploid males, haploid females and diploid tetrasporophytes, and how these factors could influence natural haploid-diploid ratios.

## Materials and methods

### Field surveys

The red macroalga *Gracilaria chilensis* is common along the Chilean shore. Individuals, fixed by a holdfast, inhabit the intertidal rocky bottom and may survive and re-grow new fronds after losing old ones [25]. Its complex life cycle alternates between isomorphic free living tetrasporophytes (diploids) and gametophytes (haploids) and is commonly named “isomorphic biphasic” or “haploid-diploid” (Fig. 1). Red algae life cycle also includes the grow of the carposporophyte after syngamy, a diploid structure that is sometime referred to as a third diploid phase and some authors refer to their life cycle as “triphasic”. However, since carposporophytes are short-lived and dependent on the haploid female thallus for development [35], within the present work we refer to our model species life cycle as biphasic or haploid-diploid, as commonly done in previous studies [4, 7, 10–15, 18–20, 25, 39–43]. In *G. chilensis,* tetrasporophyte adults produce haploid tetraspores after meiosis and release them to the environment. Settled tetraspores, germinate and grow into adult haploid male or female gametophytes. The gametophyte males release gametes and fertilization occurs on female individuals. From the fertilized oogonia, a short-lived diploid epiphytic stage (the carposporophyte, Fig. 1d) develops on the female gametophyte, producing thousands of diploid spores (i.e., carpospores, Fig. 1e) [35]. The carpospores are released into the environment where they settle, germinate and grow into diploid tetrasporophyte adults.

Demographic monitoring of individuals was performed in five intertidal rock-pools (‘Corral 1’, ‘Corral 2’, ‘Niebla 1’, ‘Niebla 2’ and ‘Niebla 3’) within two sites (Corral 39°52′27″S / 73°24′02″ W and Niebla 39°55′47″S / 73°23′57″W) along the margins of the Valdivia River estuary, from October 2009 to February 2011 at four-month intervals. In the Southern Hemisphere, the interval between the February and June, June and October, and October and February census mostly corresponds to the autumn, winter and spring and summer, respectively. All individuals within each rock-pool were mapped relative to a pair of fixed points (see Engel et al. [11]). A small fragment of the thallus was collected from each individual for the identification of reproductive males (M), females (F) and tetrasporophytes (D for diploids) by their reproductive structures under a binocular microscope. Vegetative individuals, were identified using the sex-specific molecular markers developed in Guillemin et al. [25]. At each census, frond length and diameter were recorded for each observed individual. The volume (v, in cm^3^) of a cylinder of equal length and diameter was used as a proxy for ramet biomass since this estimate correlated with dry weight (r^2^ = 0.877; *P* < 0.0001; *n* = 281). Every individual absent after 4 months was re-checked after 8 months for confirmation and considered dead when absent in the re-check.

### Probability of becoming fecund (ρ)

**Fig. 2:**
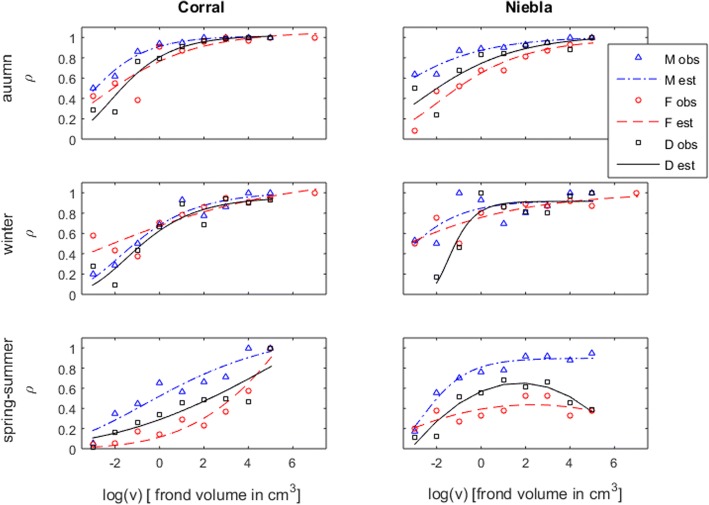
Probability of an individual of *Gracilaria chilensis* to be fecund (ρ) across different frond size categories (ln(v)). Labels are (M) males, (F) females, and (D) diploids, (obs.) observed and (est.) estimated. Individual sizes are given by the frond volume (v) in cm^3^. Gompertz curves fitted by the Newton-Rahpson method and confirmed by Levenberg-Marquardt algorithm. The probabilities of M, F and D curves to be equal, as estimated by χ2 tests, were always below 0.0002

The observed probability of an individual to become fecund (ρ_obs_) was inferred for each site, season and size class by ρ_obs_ = n_f_/n_t_, where n_f_ was the count of fecund individuals and n_t_ the total count of individuals. Nine size classes were defined: ln(v) < − 1.5, seven equally spaced classes within − 1.5 < ln(v) < 5.5, and 5.5 < ln(v); where v was the frond volume in cm^3^. The ρ_obs_ were fitted to a Gompertz curve of the form ρ_est_ = K_f_∙exp.(−b_f_∙exp.(−c_f_x)), where K_f_ is the asymptotic maximum, b_f_ is the displacement of the curve along the x-axis and c_f_ is the increment rate (Fig. 2). The parameters K_f_, b_f_ and c_f_ were estimated by vertical least squares regression. However, because the least squares have no closed form (i.e, analytical) solution for the Gompertz curve, it required numerical minimization by the Newton-Rahpson method or Levemberg-Marquardt algorithm. Its detailed application is presented in Additional file 1. A total of 18 Gompertz curves (i.e., three “seasons” × two sites (Niebla and Corral) × 3 stages (haploid females, haploid males and diploid tetrasporophytes)) were estimated describing the size-dependent fecundity (Fig. 2). Their goodness-of-fit was estimated from χ^2^ tests with the significance corresponding to the probability of the curves not fitting the observations. With 7 ≤ *i* ≤ 9 size classes and j = 3 parameters needing estimation, there were i-j-1 degrees of freedom. These curves were compared among life cycle stages, at each site and season. Their pair-wise resemblances were estimated from χ^2^ tests of homogeneity with the significance corresponding to the probability of two curves matching. With 7 ≤ i ≤ 9 size classes and j = 2 stages being compared, there were (i-1)(j-1) degrees of freedom.

### Spore production rates

The number of spores produced per unit frond volume was estimated for all mature female gametophytes and diploid tetrasporophytes. For each mature female gametophyte, the number of cystocarps (i.e., the short-lived diploid epiphytic stage growing on haploid female thallus) found on the 10-cm distal extremity of three branches was counted [11]. Four hundred cystocarps were sampled from 20 mature female gametophytes chosen randomly (i.e., 20 from each female) and these 400 cystocarps were placed together in 30 mL of filtered seawater in a Falcon tube kept at 4 °C. Note that a small part of the female thallus was excised jointly with the cystocarps. The number of diploid carpospores produced per cystocarp was counted after 24 h under a binocular microscope (i.e., mean over 10 independent counting of seawater drops of 50 μL). In the same way, 80 cm of mature thallus were cut from 20 random diploid tetrasporophytes (i.e., 4 cm from each tetrasporophyte) and these 80 thallus fragments were placed together in 30 mL of filtered seawater in a Falcon tube kept at 4 °C. The number of haploid tetraspores produced per centimeter of tetrasporophyte thallus was counted after 24 h under a binocular microscope (i.e., mean over 10 independent counting of seawater drops of 50 μL). Measures of the number of carpospores released per cystocarp and tetraspores released per centimeter of tetrasporophyte thallus were performed independently for Corral and Niebla and implemented after each sampling except in October 2009. For each haploid female gametophyte, the number of spores produced (p_carp_) was inferred by multiplying the average number of diploid carpospores released per cystocarp by the average number of cystocarps per cm^3^ of thallus (assuming a branch diameter of 1 mm) and by the total volume of the female individual. For each diploid tetrasporophyte, the number of spores produced (p_tet_) was inferred by multiplying the average number of haploid tetraspores released by cm^− 3^ of thallus (assuming a branch diameter of 1 mm) by the total volume of the individual. Furthermore, tetraspores could be male (Mtet) or female (Ftet). We assumed an even sex ratio and thus p_Mtet_ = p_Ftet_ = p_tet_/2.

### Spore survival rates

The spore survival probabilities were estimated from eq. (1), where ‘spore’ holds for diploid carpospores (carp), haploid male tetraspores (Mtet) and haploid female tetraspores (Ftet). This estimation required the production of the respective spores (p_spore_) at time t and in each site (Corral or Niebla). We assumed that the spores produced at each site spread evenly among its respective pools. Therefore, each pool received a fraction of the spores produced that was equal to its relative area rA_pool_ = A_pool_/A_site_, where A is area. The spores that survived to time t + 1 were detected as recruits of the respective stage (r_stage_) in the following census.

1 $$ {s}_{spore}=\frac{r_{stage}\cdot \kern0.5em {rA}_{pool}}{p_{spore}} $$

The dependency of spore survival from the local frond density was fit to a second-degree polynomial using the Iterative Reweighted Least Squares (IRLS) estimation method. The local frond density (V_p_) was quantified by the sum of all frond volumes at each pool, yet, excluding the larger frond to avoid bias from exceptionally large fronds [15, 46]. The frond density was dependent on location and season. Since they were not numerically orthogonal to the factor density and did not increase the explanatory power of the model, the effects of these factors were not tested. Nevertheless, it was possible to compare between Corral and Niebla sites. The goodness-of-fit was estimated from χ^2^ tests with the significance corresponding to the probability of the curves not fitting the observations. The number of observations were *i* = 2 pools× 9 census at Corral, *i* = 3 pools× 9 census at Niebla and j = 3 parameters needed to be estimated, resulting in 14 = i-j-1 degrees of freedom for Corral and 23 = i-j-1 degrees of freedom for Niebla.

### Correlation between vital rates and field abundances

We tested for the relation between the ploidy ratio of the abundances (i.e., the haploid-diploid ratio or H:D) and the ploidy ratio of the vital rates, the latter ratio requiring estimation according to the ploidy to which each vital rate is contributing. Thus, vital rates contributing to the haploids (production and survival of haploid tetraspores) must appear in the numerator whereas vital rates contributing to the diploids (production and survival of diploid carpospores) must appear in the denominator (see [40–43]). Hence, the ratios used to compare these rates and relate them to the H:D were the p_tet_:p_carp_, the s_tet_:s_carp_ and the R_H_:R_D_.

The direct use of the haploid-diploid ratio when testing and comparing the effects of its driving factors is inadequate because the addition of x haploid fronds to the numerator or of x diploid fronds to its denominator do not have symmetrical effects. Hence, the haploid-diploid ratio inevitably shows widely different sensitivities and elasticities to ecologically equivalent vital rates just because these contribute to different sides of the ratio. For this same reason the haploid-diploid ratio of a species cannot follow a normal distribution (or any symmetrical distribution for that matter) particularly when there is change in phase dominance among locations or seasons. Since statistical tests using the haploid-diploid ratio as dependent (response) variable are inadequate, the haploid-diploid ratio was replaced by the log(H:D) [40, 43, 47]. The point of such procedure is exemplified in the following text. Consider the situation (H:D_0_) with the abundances balanced at 1:1, the alternative H:D_A_ unbalanced at 10:1 and the alternative H:D_B_ unbalanced at 1:10. The direct use of the haploid-diploid ratio metric falsely suggests that the alternative A represents a change (A-0) of magnitude 9 whereas the alternative B represents a change (B-0) of magnitude 0.9. An honest comparison between the alternatives A and B requires the use of logarithms (e.g., [40, 43, 47]). In this example log_10_(1:1) = 0, log_10_(10:1) = 1 and log_10_(1:10) = − 1, revealing that the alternatives A and B represent changes of equal magnitude but opposite directions. The statistic log(H:D) may perfectly well follow a normal distribution without restrictions about which phase is dominant. Hence, in this work the haploid-diploid ratio of abundances and vital rates were always tested and plotted in logarithmic scales.

## Results and discussion

### Probability of fronds to become fecund

The probability of fronds becoming fecund (ρ) was size-dependent, with larger fronds generally fecund (i.e., ρ ≈ 1), and well fitted by Gompertz curves (Fig. 2). The exceptions were the haploid female gametophytes and the diploid tetrasporophytes at Niebla during the spring-summer. Their decreasing ρ was better fitted by quadratic functions. All these curves fitted the observations remarkably well (χ^2^ *p* < 0.007). Fecundity was highly seasonal: during the austral autumn and winter most fronds became fecund, even the small. On the other hand, the austral spring-summer was less favorable for becoming fecund. At Corral, many of the small and medium sized fronds - particularly of the haploid females and diploids - were not becoming fecund. At Niebla, even the large haploid females and diploids were not becoming fecund. The ρ was dependent on life cycle stage, with advantage for the haploid males. The χ^2^ tests showed that the Gompertz curves estimated for each stage were always different (χ^2^ *p* < 0.0002). However, during the autumn and winter, the differences among stages were mostly restricted to the smaller fronds and only during the spring-summer did they generalize to the full frond size range. This contrast among stages may represent a contrast between strategies for resource allocation in times of shortage or intense competition; with small and medium sized diploids and haploid females investing in survival, and haploid males investing in fecundity. Furthermore, our companion study about *G. chilensis* survival [15] revealed a low survival of males when compared to females and diploids, especially for small individuals. In fact, the haploid female (F), haploid male (M) and diploid tetrasporophyte (D) differentiation of survival [15] was perfectly symmetric to their differentiation of becoming fecund (ρ). This mirroring pattern between probabilities of survival and becoming fecund support the existence in the field of a trade-off between survival and reproduction [48–50] that had already been reported for *G. chilensis* reared in the laboratory [51]. Within the long list of theoretical works exploring potential differences between phases in haploid-diploid organisms, Lewis [52] proposed that, when resources are scarce, haploids might benefit from having half the cost of production and upkeep of DNA than diploids. Lewis theory has been further developed into the “nutrient limitation hypothesis” [53] and this non-genetic explanation for the evolution of life cycles has been sustained by experimental studies showing that haploids grew faster than diploids when cultivated in nutrient-poor conditions [23]. Resources become scarce under intense competition or when the adaptations required to overcome hydrodynamic stress, desiccation or herbivory are resource demanding. In all such cases the *G. chilensis* haploid females survived better than any other stage [15] while the haploid males had greater probability of becoming fecund than any other stage (this study). It is possible that resources saved in haploids by producing half the DNA are differently allocated depending on sex: to ensure a better survival in female and a higher fecundity in males.

### Fecundity

**Fig. 3:**
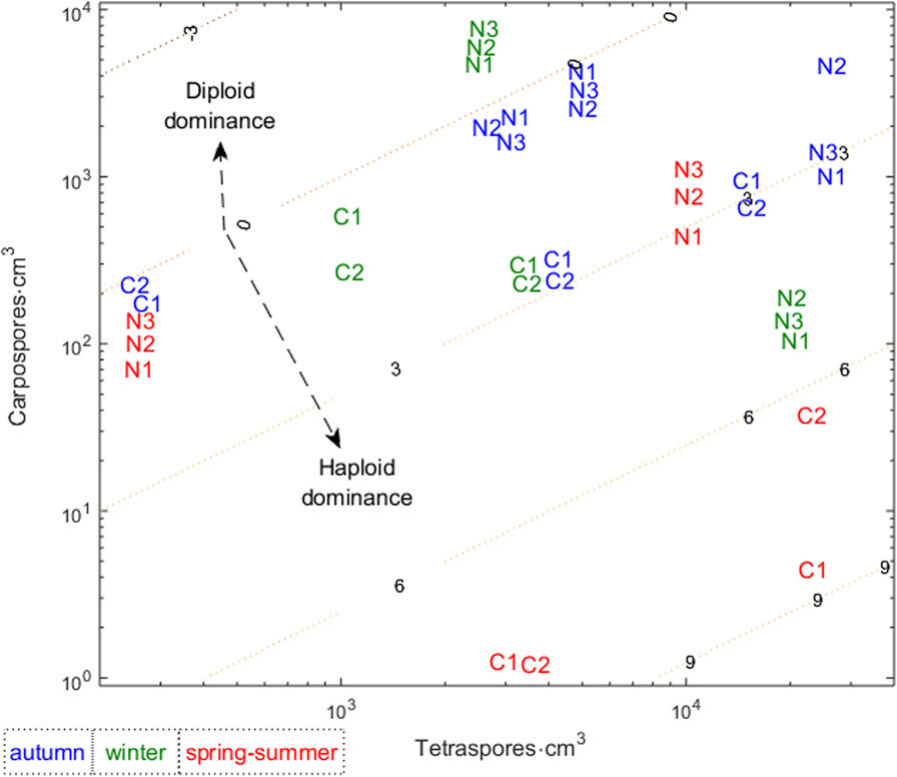
Tetra- and carpospore production rates of *Gracilaria chilensis.* Spores per cm^3^ of parental thallus observed during three seasons in five intertidal pools at sites (C) Corral and (N) Niebla. Contour lines represent log(p_tet_/p_carp_). Because the study lasted almost 3 years, some seasons show 2 replicates while others show 3 replicates

Besides the differentiation of the probability of becoming fecund (ρ), a differentiation of fecundity itself (i.e., the production of spores per cm^3^ of thallus) was observed between haploid females and diploids. The 3-way permutation test with 1000 randomizations determined that the difference among stages, among sites and among seasons were all significant, as was the stage×season interaction (Table 1). Tetrasporophytes produced an average of 7939 tetraspores (haploid spores) per cm^3^ of frond whereas the female gametophytes produced an average of 1406 carpospores (diploid spores) per cm^3^. Average tetraspore production per individual was 6.97 times higher than carpospore production. This dominance of haploid spores production matches those previously reported in haploid-diploid algae and was argued to be one of the probable causes of dominance of haploid gametophytes in natural stands [12, 38, 39]. During the austral spring-summer fewer carpospores were produced than during the other seasons, resulting in a more tetraspore dominated spore production (Fig. 3). Overall, the summer was the season with the lowest *G. chilensis* fecundity: fronds were less likely to become fecund and the fecund fronds produced fewer spores. Relative to the factor ‘Site’, much more spores were produced at the Niebla pools than at the Corral pools (Fig. 3). Since, the fronds at Corral dried on the flat bare rock during low tide, it is possible that the stress from desiccation at this site may demand considerable resource allocation to survival therefore enhancing trade-off between survival and fertility.

**Table 1:** Three-way ANOVA on the spore production of *Gracilaria chilensis* across the stages haploid females, haploid males and diploids; the seasons autumn, winter and spring-summer; and the pools Corral 1, Corral 2, Niebla 1, Niebla 2 and Niebla 3

Factor	effect	d.f.	F	*p*
Stage	Fixed	1	37.957	**< 0.001**
Season	Fixed	2	8.157	**0.005**
Site	Fixed	4	3.103	**0.039**
Stage×season	Fixed	2	4.902	**0.007**
Stage×site	Fixed	4	1.714	0.154
Season×site	Fixed	8	0.057	0.999
Stage×site×season	Fixed	8	1.525	0.186

The dependent variable ‘spore production’ was log-transformed to ascertain the homogeneity of variances within groups. The d.f._error_ = 40. Significant *p*-values shown in **bold**

### Spore survival

**Fig. 4:**
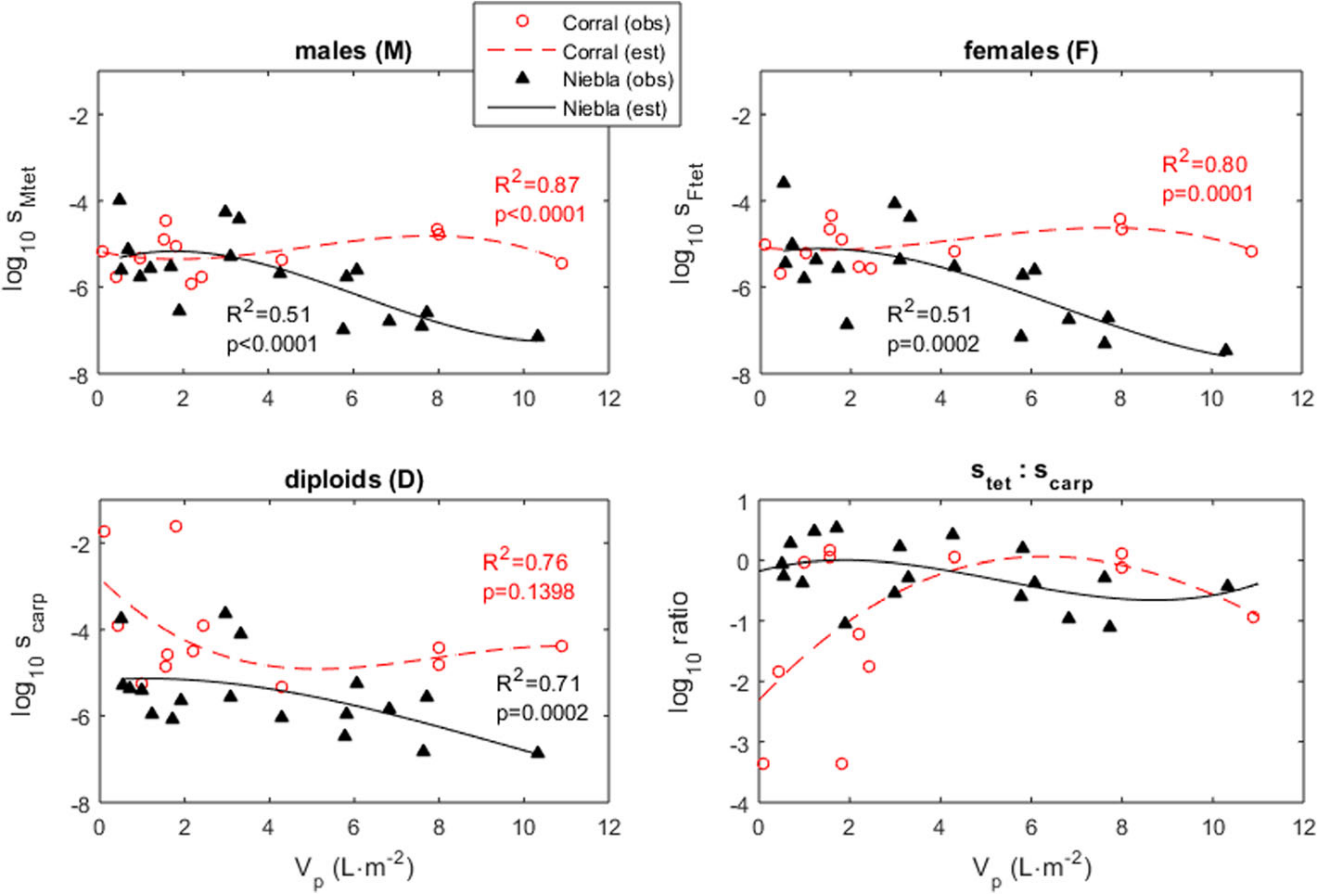
Spore survival rates of *Gracilaria chilensis*. Labels are (obs.) observed and (est.) estimated. Pool densities are given by their total frond volume (V_p_) excluding the larger frond. Also shown are the non-linear coefficients of determination (R^2^) and Pearson’s χ2 goodness-of-fit test (p)

A pattern of differentiation of spore survival among life cycle stages common to all algae, or even all Gracilariales, was impossible to find so far. Studies have shown that advantages toward carpospores (diploids) or tetraspores (haploids) for settlement and germination varied depending on species, season or environmental conditions [13, 18, 23–25, 36, 37, 54, 55]. Since our census was done at four-month intervals, the estimation of spore survival included all the processes taking place during this time frame, namely their survival while drifting, settlement, germination and the survival of the sporelings. Despite the coarse temporal resolution of our data, the second degree polynomials were able to clearly identify the sporelings’ survival dependency on the local frond density (Fig. 4). Posterior simulations demonstrated that using these polynomials resulted in good fits to the observed haploid:diploid spore survival ratios (s_tet_:s_carp_) (Fig. 4). This ratio was mostly beneficial to the diploids, particularly at the Corral site and under the lowest frond densities (Fig. 4). In the following paragraphs we detail this spore survival dynamics and raise the hypothesis that maternal care may be at the origin of the diploid advantage. In fact, a generalized haploid advantage over the adult fronds of stressed intertidal red algal stands has been reported [11, 18, 21, 29, 44, 56–58] while a generalized diploid advantage was observed for sporelings [23, 25, 44], suggesting that maternal care by the haploid females protecting their carpospore progeny in adverse environments could be a generalize tendency in red macroalgae.

Our results showed the negative effect of high frond densities on the survival of the spores and sporelings. The high density – low survival relation is well documented in algal species as a consequence of intraspecific competition, leading to the famous self-thinning rule [59, 60]. This negative effect has been reported in *G. chilensis* [15] and has even been observed between sporelings in other seaweed species [61]. During our survey, at high frond densities, the increased mortality of settled spores was pronounced at the Niebla site for all spore types, while at the Corral site it was restricted to the haploid tetraspores (Fig. 4). As said above, even though both survey sites where located in the upper intertidal, they differ in terms of desiccation and UV stress levels during low tide. While, Niebla rock pools retain seawater during low tide protecting fronds from severe dehydration, the fronds at Corral dried on the flat bare rock. We propose that, at Corral, the positive effect of protection against desiccation and UV stress provided by the adult fronds override the negative effect of competition unless the stands were outstandingly crowded. Interestingly, we observed that whenever spore survival depended on competition with adult fronds (i.e., at high frond density), the carposporelings (diploids) outperformed the tetrasporelings (haploids). This advantage of the diploid over the haploid sporelings contrasts with results showing the advantage of *G. chilensis* adult haploid female fronds over the diploid fronds under the same stress from competition [15]. These results could suggest the existence of maternal care by the haploid females. Indeed, females, by providing nutrients to their carpospores progeny, can lead to an advantage of diploid carpospores over haploid tetraspores under scarcity of resources. The maternal care by the haploid females providing nutrients and thus benefiting the production, development and germination of diploid carpospores has been reported in three other red algal species [35].

Our results also showed the negative effect of low frond densities on the survival of the spores and sporelings. Low frond densities have been shown to be stressful, exposing individuals to desiccation [8, 18, 22, 62, 63] or to hydrodynamic stress [8, 18, 21, 64], affecting the survival of intertidal algae (i.e., Allee effects). Our work showed that in Corral the diploid spores and sporelings survived better than the haploid spores when growing at low frond densities (Fig. 4). These differences in survival were observed during summer for the Corral algal stands (where *G. chilensis* dry on the flat bare rock during low tide). We propose that the advantage of diploid spores under these adverse conditions could also come from maternal care by the haploid female, who can pass-on to their diploid progeny the chemical compounds conferring resistance to UV and herbivory, that the sporelings are unable to produce and accumulate on their own. Supporting our hypothesis, the better survival of the *G. chilensis* adult haploid female fronds under desiccation and UV stress was also particularly evident at Corral during the summer [15] and has been linked to a possible accumulation of chemical compounds related to photoprotection [65, 66] and antioxidative enzymes [67]. A better ability of the diploid carpospores to withstand UV radiation than the haploid tetraspores has been demonstrated in other red algae [68]. Consistently, haploid females in red algae have been reported to present higher concentrations of protective chemical compounds within cystocarps than along the main thallus [20], a pattern that suggested the existence of a mechanism of strong protection of the cystocarps and diploid carpospores.

### Factors determining the haploid:Diploid ratio of *Gracilaria chilensis* recruits

**Fig. 5:**
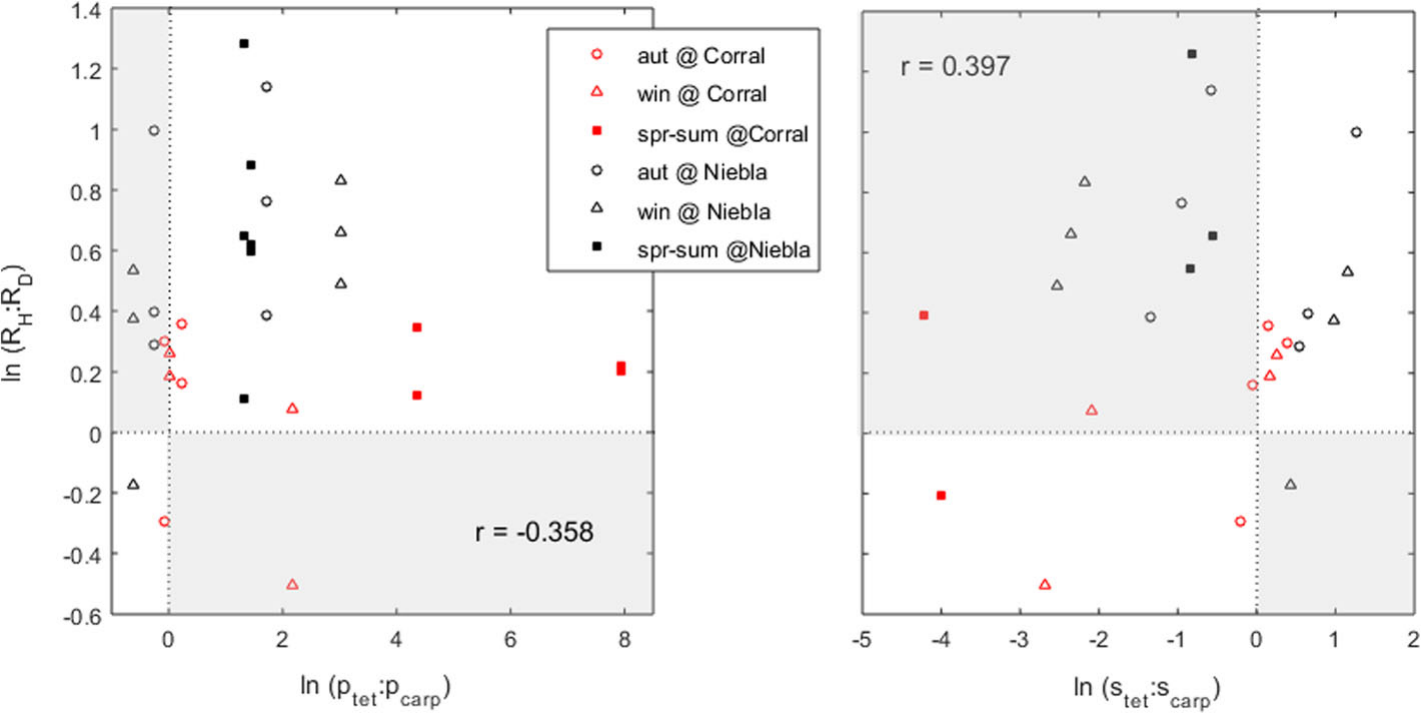
Correlation between the ploidy ratios of *Gracilaria chilensis* spore vital rates at time t and the ploidy ratios of recruitment at time t + 1. Labels are (s) survival, (p) production, (R) recruitment, (ln) Neper’s logarithm, (carp) carpospores, (tet) tetraspores, (H) haploids, and (D) diploids. Grey areas represent incompatibility between the ploidy ratios of the spore vital rates and the ploidy ratios of the recruitment

Recruitment is fundamental for the determination of the future population structure. However, recruitment itself is an intricate process resulting from the interaction of spore production, survival, settlement, germination and the development of the sporelings. While spore production has been proposed as the fundamental driver of the ploidy ratio of abundances [12, 38, 39], others have argued that settlement and germination rates are more important factors [36, 37]. Concomitantly with the ranking of such rates, shifts in ploidy dominance are commonly found when moving from spore production to spore settlement and germination [13, 14, 36, 37]. The present study shows that in *G. chilensis* the production of tetraspores (haploids) is usually higher than of carpospores (diploids), whereas the survival of the diploid carposporelings dominates over the survival of haploid tetrasporelings. However, the correlations between the ploidy ratio of recruitment and the ploidy ratios of spore production or of spore survival were very weak, and none of these processes dominated the recruitment of *G. chilensis* young fronds (Fig. 5). The importance of each of these two components in explaining the ploidy ratio of the recruits depended greatly on site and season. This finding rejects previous hypothesis (i.e, [12, 36–39]) proposing that the population structure (namely, its ploidy ratio) is determined by one specific aspect within fertility. On the contrary, our results suggest that an advantage of haploids or of diploids depends on a multitude of biological processes and their differential responses to the local conditions.

### Perspective on haploid-diploid life cycle maintenance

Contrasting results about survival of the adult fronds of *G. chilensis* [15], with their fecundity and the survival of their spores (the present work) allow a more complete understanding of the species population dynamics. We propose that tetrasporophyte (diploid) fronds present a more r-oriented strategy. On the other hand, female gametophyte (haploid) fronds, seem to present a more K-oriented strategy; investing further resources in their survival, producing fewer spores, but investing in the survival of their carpospore (diploid) progeny through maternal care. The evolutionary stability of this life cycle suggests that, for this type of organisms in their habitat, complementary ploidy phases with different life strategies has more success than a monophasic alternative.

## Additional files

Additional file 1: Gompertz. (DOCX 39 kb)

Additional file 2: All.mat. (MAT 64 kb)

Additional file 3: Weibull. (DOCX 78 kb)

## Abbreviations

ANOVA: Analysis of Variance
C: Corral sampling site
C1: Corral pool #1
C2: Corral pool #2
carp: Carpospores
D: Diploids
DNA: Deoxyribonucleic acid
F: Female haploids
F_tet_: Female tetraspores
G: Gametophytes
G:T: Gametophyte-to-tetrasporophyte ratio
H: Haploid
H:D: Haploid-to-diploid ratio
IRLS: Iterative Reweighted Least Squares
M: Male haplois
M_tet_: Male tetraspores
N: Niebla sampling site
N1: Niebla pool #1
N2: Niebla pool #2
N3: Niebla pool #3
p_tet_:p_carp_: Ratio of tetraspore-to-carpospore production
R_H_:R_D_: Ratio of haploid-to-diploid recruits
RNA: Deoxyribonucleic acid
s_tet_:s_carp_: Ratio of tetraspore-to-carpospore survival
T: Tetrasporophytes
tet: Tetraspores
UV: Ultra-violet radiation
