# Supplementary material for: Differentiation of haploid and diploid fertilities in Gracilaria chilensis affect ploidy ratio
Source: BMC Evol Biol. 2018 Dec 5;18:183. doi: 10.1186/s12862-018-1287-x (PMC6280411; doi:10.1186/s12862-018-1287-x)
Supplement: Supplementary file 1 — Gompertz. (DOCX 39 kb) [file 12862_2018_1287_MOESM1_ESM.docx]

The probability of any individual sized x=ln(v) to be fecund followed a Gompertz curve, where K_f_ is the asymptotic maximum, b_f_ is the displacement of the curve along the x axis, and c_f_ is the increment rate:

$\rho_{est}=K_{f}\cdot e^{-b_{f}\cdot e^{-c_{f}\cdot x}}$ (1)

The parameters β={K_f_,b_f_,c_f_} were estimated by vertical least squares regression. Because it has no closed form (i.e, analytical) solution for the Gompertz curve, these least squares required numerical minimization:

$\left\{K_{f},b_{f},c_{f}\right\}=\mathop{\mathrm{argmin}}\limits_{\beta} \sum\left(\rho_{obs}-\rho_{est}\right)^{2}$ (2)

The ∑(ρ_obs_-ρ_est_)^2^ is the Sum of Squares of the Error (SSE). When it is minimized, its derivative (∂SSE/∂β) is zero. In this case we had a function with three unknowns, hence three partial derivatives that all needed to converge to zero. The Newton-Raphson method iteratively converged to the roots of the ∂SSE/∂β estimating:

$\beta_{t+1}=\beta_{t}-\frac{\frac{\partial SSE}{\partial\beta}}{\frac{\partial^{2}SSE}{\partial\beta^{2}}}$ (3)

Its demonstration is beyond of the scope of this Appendix. Its application required the first and second order partial derivatives estimated as:

$\frac{\partial SSE}{\partial\beta}=\left\{\begin{matrix} \frac{\partial SSE}{\partial K_{f}} \\ \frac{\partial SSE}{\partial b_{f}} \\ \frac{\partial SSE}{\partial c_{f}} \end{matrix}\right\}$ (4)

$\frac{\partial SSE}{\partial K_{f}}=-2\sum\left(\rho_{obs}\cdot e^{-b_{f}\cdot e^{-c_{f}}}-K_{f}\cdot e^{-2b_{f}\cdot e^{-c_{f}\cdot x}}\right)$ (5)

$\frac{\partial SSE}{\partial b_{f}}=2\sum\left(\rho_{obs}\cdot K_{f}\cdot\exp^{-c_{f}\cdot x-b_{f}\cdot e^{-c_{f}.x}}-K_{f}^{2}\cdot e^{-c_{f}\cdot x-2b_{f}\cdot e^{-c_{f}\cdot x}}\right)$ (6)

$\frac{\partial SSE}{\partial c_{f}}=-2\sum\left(\rho_{obs}\cdot K_{f}\cdot b_{f}\cdot x\cdot e^{-c_{f}.x-b_{f}\cdot e^{-c_{f}\cdot x}}-K_{f}^{2}\cdot b_{f}\cdot x\cdot e^{-c_{f}.x-2b_{f}\cdot e^{-c_{f}\cdot x}}\right)$ (7)

$\frac{\partial^{2}SSE}{\partial\beta^{2}}=\left\{\begin{matrix} \frac{\partial^{2}SSE}{\partial K_{f}^{2}} & \frac{\partial^{2}SSE}{\partial K_{f}\partial b_{f}} & \frac{\partial^{2}SSE}{\partial K_{f}\partial c_{f}} \\ \frac{\partial^{2}SSE}{\partial b_{f}\partial K_{f}} & \frac{\partial^{2}SSE}{\partial b_{f}^{2}} & \frac{\partial^{2}SSE}{\partial b_{f}\partial c_{f}} \\ \frac{\partial^{2}SSE}{\partial c_{f}\partial K_{f}} & \frac{\partial^{2}SSE}{\partial c_{f}\partial b_{f}} & \frac{\partial^{2}SSE}{\partial c_{f}^{2}} \end{matrix}\right\}$ (8)

$\frac{\partial^{2}SSE}{\partial K_{f}^{2}}=2\sum\left(K_{f}\cdot e^{-2b_{f}\cdot e^{-c_{f}\cdot x}}\right)$ (9)

$\frac{\partial^{2}SSE}{\partial K_{f}\partial b_{f}}=\frac{\partial^{2}SSE}{\partial b_{f}\partial K_{f}}=2\sum\left(\rho_{obs}\cdot e^{-c_{f}-b_{f}\cdot e^{-c_{f}}}-2K_{f}\cdot e^{-c_{f}-2b_{f}\cdot e^{-c_{f}\cdot x}}\right)$ (10)

$\frac{\partial^{2}SSE}{\partial K_{f}\partial c_{f}}=\frac{\partial^{2}SSE}{\partial c_{f}\partial K_{f}}=-2\sum\left(\rho_{obs}b_{f}xe^{-c_{f}x-b_{f}e^{-c_{f}x}}-2K_{f}b_{f}xe^{-c_{f}x-2b_{f}\cdot e^{-c_{f}\cdot x}}\right)$ (11)

$\frac{\partial^{2}SSE}{\partial b_{f}^{2}}=-2\sum\left(\rho_{obs}\cdot K_{f}\cdot e^{-2c_{f}\cdot x-b_{f}\cdot e^{-c_{f}\cdot x}}-2K_{f}^{2}\cdot e^{-2c_{f}\cdot x-2b_{f}\cdot e^{-c_{f}\cdot x}}\right)$ (12)

$\begin{matrix} \frac{\partial^{2}SSE}{\partial b_{f}\partial c_{f}}=\frac{\partial^{2}SSE}{\partial c_{f}\partial b_{f}}=2\sum\left(\rho_{obs}K_{f}e^{-c_{f}x-b_{f}e^{-c_{f}x}}\cdot x\cdot\right.\left(b_{f}\cdot e^{-c_{f}\cdot x}-1\right)+ \\ \left.-K_{f}^{2}\cdot e^{-c_{f}x-2b_{f}\cdot e^{-c_{f}\cdot x}}\cdot x\cdot\left(1-2b_{f}\cdot e^{-c_{f}\cdot x}\right)\right) \end{matrix}$ (13)

$\begin{matrix} \frac{\partial^{2}SSE}{\partial c_{f}^{2}}=-2K_{f}\cdot b_{f}\sum\left(\rho_{obs}\cdot e^{-c_{f}x-b_{f}e^{-c_{f}x}}\cdot x^{2}\cdot\right.\left(b_{f}\cdot e^{-c_{f}\cdot x}-1\right)+ \\ \left.-K_{f}\cdot e^{-c_{f}x-2b_{f}\cdot e^{-c_{f}\cdot x}}\cdot x^{2}\cdot\left(2b_{f}\cdot e^{-c_{f}\cdot x}-1\right)\right) \end{matrix}$ (14)

The Newton-Raphson method in its original form is prone to numerical instability, and its application to the estimation of the Gompertz curve parameters is one such case. In order to stabilize it, we adopted a simple solution of restraining the size of the step taken towards the roots. The Newton-Raphson method became:

$\beta_{t+1}=\beta_{t}-\frac{\frac{\partial SSE}{\partial\beta}}{\frac{\partial^{2}SSE}{\partial\beta^{2}}}\wedge\left|\frac{\frac{\partial SSE}{\partial\beta}}{\frac{\partial^{2}SSE}{\partial\beta^{2}}}\right|\leq\left|0.2\beta\right|$ (15)
