# Supplementary material for: Differentiation of haploid and diploid fertilities in Gracilaria chilensis affect ploidy ratio
Source: BMC Evol Biol. 2018 Dec 5;18:183. doi: 10.1186/s12862-018-1287-x (PMC6280411; doi:10.1186/s12862-018-1287-x)
Supplement: Supplementary file 3 — Weibull. (DOCX 78 kb) [file 12862_2018_1287_MOESM3_ESM.docx]

The probability of any individual sized *x*=ln(*v*) to be fecund followed a Weibull curve, where *K_∞_* was the upper asymptote, *K_0_* was the lower asymptote, *b* was the growth rate and *c* set the displacement along the *x* axis.

 (1)

However, the Weibull function does not accept x<0; meaning that, whenever x<0 the φ_i_ entered the domain of complex numbers (i.e, with an imaginary component). But even if always x>0, the optimization algorithms used for the parameter estimation tend to become numerical unstable when at least *x* or *y* (in this case *y*=*φ*) is widely >1. Hence, these were both transformed to vary within 0<*ẋ*<1 (equation 2a) and 0< *ẏ* <1 (equation 2b). The *x_max_* corresponded to the maximum observed value of *x*. However, *x_min_* had to be a little below the minimum observed value of *x* so that its corresponding *ẋ*>0. Being this minimum a negative value, we set *x_min_*=1.1×min(*x*). This constrain arose from the Newton-Raphson Method used for the parameter estimation requiring first and second order partial derivatives, which in the case of the Weibull function (or of any of its similar) include log(*ẋ*). Hence, if *ẋ*=0, the log(0)=-∞ and the parameter estimation “blew” on our faces. The *φ_max_* corresponded to the maximum observed value of *φ* whereas the *φ_min_* had to be a little below the minimum observed value of *φ* so that its corresponding *ẏ*>0.

 (2a) (2b)

The parameters *β*={*K_∞_*,*K_0_*,*b*,*c*} were estimated by vertical least squares regression. Because it has no closed form (i.e, analytical) solution for the Weibull curve, these least squares required numerical minimization (eqn. 3). The ∑(*φ_obs_*-*φ_est_*)^2^ is the Sum of Squares of the Error (*SSE*). When it is minimized, its derivative (∂*SSE*/∂*β*) is zero. In this case we had a function with four unknowns, hence four partial derivatives that all needed to converge to zero. The Newton-Raphson method iteratively converged to the roots of the ∂*SSE*/∂*β* (eqn.4).

 (3) $\beta_{t+1}=\beta_{t}-\frac{\frac{\partial SSE}{\partial\beta}}{\frac{\partial^{2}SSE}{\partial\beta^{2}}}$ (4)

Its application required the first and second order partial derivatives arranged in the (4×1) vector and (4×4) matrix:

 (5) (6)

Their entries required the observed and predicted values of ẏ as well as the ∂ẏ∂β_i_ evaluated at the corresponding ẋ:

 (7)

 (8)

 (9)

 (10)

 (11)

 (12)

 (13)

 (14)

 (15)

 (16)

 (17)

 (18)

 (19)

 (20)

 (21)

 (22)

 (23)

 (24)

 (25)

 (26)

 (27)

 (28)

 (29)

 (30)

 (31)

 (32)

 (33)

 (34)

Although the intended model application included previously transforming *x* into *ẋ*, it still required the φ output. Hence, the β_i_ estimated for ẏ needed to be converted into the β_i_ required by φ:

 (35)
